# Cerebral Venous Sinus Thrombosis Following Varicella Infection: A Case Report

**DOI:** 10.7759/cureus.72448

**Published:** 2024-10-26

**Authors:** Mohammed Dablouk, Ahmed Musa

**Affiliations:** 1 Internal Medicine, Mersey and West Lancashire Teaching Hospitals NHS Trust, Whiston, GBR; 2 General Practice, Swansea Bay University Health Board, Swansea, GBR

**Keywords:** anticoagulation, cerebral venous sinus thrombosis (cvst), headache, thrombus, varicella zoster (chicken pox)

## Abstract

A 38-year-old man presented to the emergency department with a severe frontal headache, which began three days prior without visual, speech, or balance disturbances. His past medical history was unremarkable, apart from raised cholesterol. He confirmed a recent primary Varicella-zoster virus infection (chicken pox) two weeks prior. Clinical examination revealed crusted varicella lesions on the arms, trunk, and thighs. The neurological examination revealed no deficits, neck rigidity, or abnormal gait. Routine blood investigations were unremarkable. Autoantibody screen and HIV serology were negative. CT head non-contrast showed evidence of hyperdense bilateral transverse sinus thrombosis. To confirm the findings, a CT venogram showed extensive and occlusive left transverse and sigmoid sinus thrombosis with further extension into the left internal jugular vein. The stroke team advised an MRI of the head, which reported no acute infarction, and magnetic resonance venography (MRV), which further confirmed the occlusion in the left transverse sinus, sigmoid sinus, and jugular vein. Hematology was involved and advised to start warfarin and bridging therapy with enoxaparin. His migraines experienced a substantial improvement within 48 hours of commencing treatment. He was subsequently discharged with outpatient follow-up. He continued on warfarin with a therapeutic international normalized ratio (INR) range of two to three for one year. A thrombophilia screen, *JAK2*, and lupus anticoagulant were checked by hematology as part of outpatient investigations. During the first six months, he experienced mild intermittent headaches; however, for the following six months, his symptoms ultimately resolved. Following a clinic evaluation one year later, his warfarin was discontinued.

## Introduction

Varicella-zoster virus (VZV), a member of the *Herpesviridae* family, is the causative agent of chicken pox (varicella) and shingles (herpes zoster). VZV infection primarily occurs in childhood, manifesting as chicken pox, which is characterized by a pruritic vesicular rash, fever, and malaise. Following the primary infection, VZV establishes latency in the dorsal root ganglia and can reactivate later in life, leading to shingles, a painful dermatomal rash often accompanied by severe neuralgia [1]. Although chicken pox is generally considered a benign and self-limiting illness in immunocompetent children, complications can arise, particularly in immunocompromised individuals and adults. These complications include bacterial superinfections, pneumonia, hepatitis, and neurological manifestations such as encephalitis, cerebellar ataxia, and stroke. Chicken pox-related neurological complications are seen in less than 1% of cases of chicken pox [2]. One rare but severe neurological complication associated with VZV infection is cerebral venous sinus thrombosis (CVST). In this case report, we present a rare instance of CVST following VZV infection in a healthy 38-year-old male who presented with a headache.

## Case presentation

A 38-year-old Indian male with a recent history of primary VZV infection (chicken pox) presented to the emergency department with a severe frontal headache. The headache began three days prior to admission; it was not associated with visual symptoms, speech impairment, or dizziness. He reported occasional nausea but without vomiting. It was localized in the frontal region with no radiation and progressively worsened, reaching maximal intensity on the day of admission. No seizures occurred. The patient reported a recent chicken pox infection; the rash started 14 days prior to admission. On presentation, widespread lesions involving his arms, trunk, and thighs were noted and had crusted.

The patient appeared well, although in obvious discomfort, with both his hands holding his head in pain. The temperature was 38.1°C, heart rate was 105 beats per minute, saturation was 96% on room air, blood pressure was 135/75, and respiratory rate was 14. The Glasgow Coma Scale (GCS) score is 15/15. The neurological examination revealed a power rating of 5/5 in both the upper and lower limbs; sensation was intact throughout; planters exhibited flexion; no visual defects were identified; no ophthalmoplegia was noted; coordination was intact; and there were no meningeal signs. The fundoscopy results were unremarkable. The electrocardiogram (ECG) indicated sinus tachycardia.

Imaging

**Figure 1:**
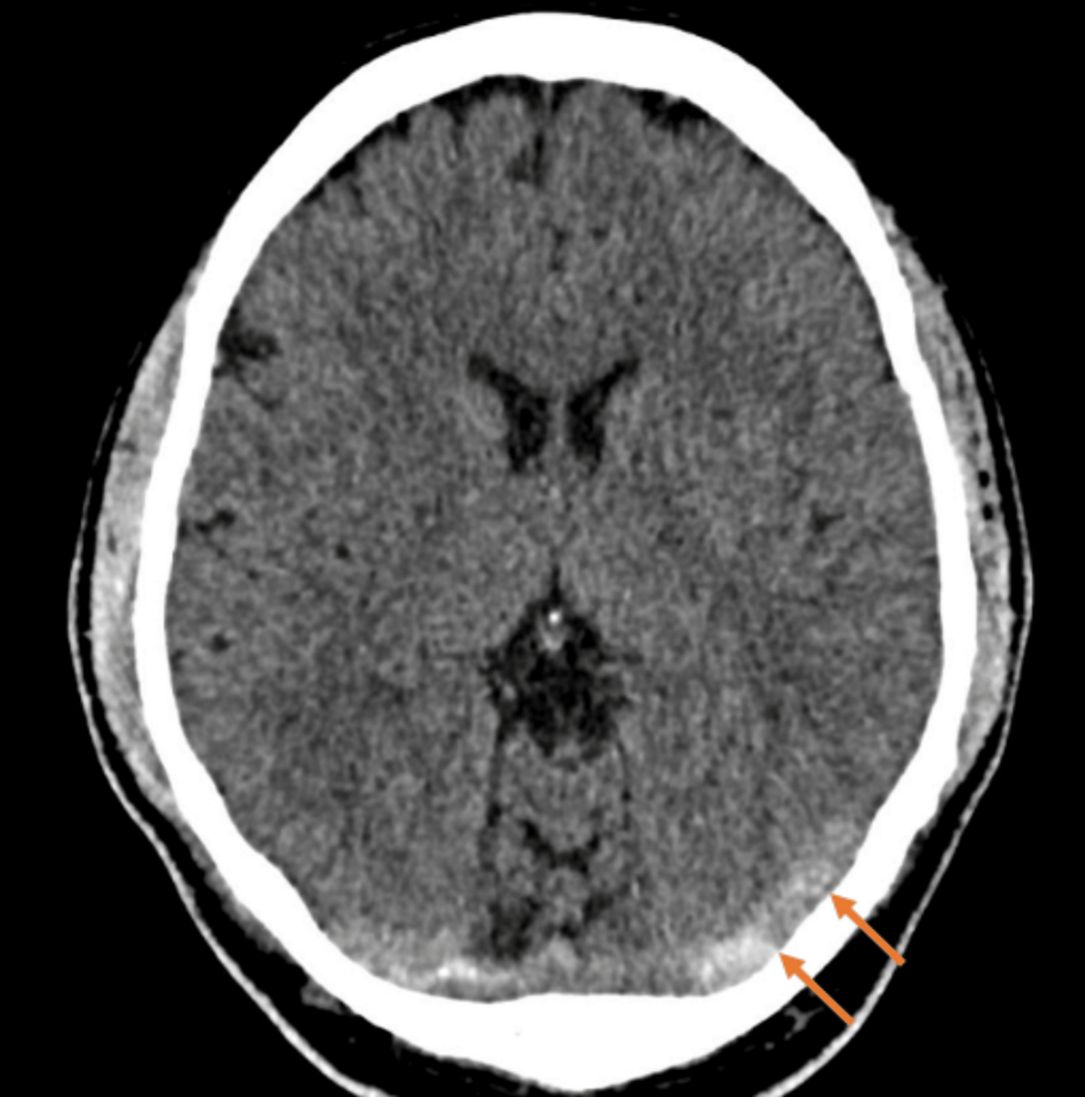
A non-enhanced CT image (axial view) showing areas of abnormal hyperattenuation (orange arrows) that are consistent with CVST in the left transverse sinus CVST: cerebral venous sinus thrombosis; CT: computed tomography

The non-contrast computed tomography (CT) of the head showed evidence of hyperdense bilateral transverse sinus thrombosis. A CT venogram (CTV) was conducted to confirm the findings, which showed extensive and occlusive left transverse and sigmoid sinus thrombosis with further extension into the left internal jugular vein (Figure 1). A magnetic resonance imaging (MRI) of the head was performed, and no acute infarction was reported. The magnetic resonance venography (MRV), however, showed that there is still occlusion with no contrast flow in the left transverse sinus, sigmoid sinus, and jugular vein. The findings from the CTV are consistent with thrombosis. There is some flow artifact in the right transverse sinus-sigmoid junction, but no definite thrombosis is present.

The patient was started on 1 mg/kg of enoxaparin twice daily for two days. During this time, his headache markedly improved. After imaging confirmation of the diagnosis, he was switched to warfarin, and his INR was monitored until the therapeutic range of 2-3. His symptoms subsided, and he felt better posttreatment, leading to his discharge from the hematology service subsequently.

## Discussion

Primary VZV infection, generally referred to as chicken pox, is a temporary febrile sickness that results in a rash characterized by fluid-filled blisters. This condition is mostly seen in children. Reactivation of VZV is more common in adults than original varicella infection. VZV may lead to several complications, such as encephalitis, cerebellar ataxia, transverse myelitis, ventriculitis, meningoencephalitis, and aseptic meningitis [3]. Additionally, it may be linked to ischemic stroke, carotid dissection, aneurysm, and subarachnoid or cerebral hemorrhages due to arterial vasculopathy. In certain cases, VZV may lead to the development of purpura fulminans and venous thrombosis due to an abnormal tendency for blood clotting [4]. The patient had a severe case of CVST, which is an uncommon complication that may occur as a result of primary VZV infection.

Virchow's trinity, consisting of vascular wall damage, hypercoagulability, and stasis, is the underlying cause of venous thrombosis [4]. The establishment of venous thrombosis in post-primary VZV infection is believed to be caused by direct injury to the endothelial cells, inflammation of the blood vessel, the production of autoantibodies against protein S, or an existing condition of increased blood clotting. In 2016, Paul et al. reported that two out of three post-varicella CVST cases were associated with protein S deficiency, a preexisting condition that causes increased blood clotting [8]. In 2012, Siddiqi and colleagues documented two instances of post-VZV CVST resulting from preexisting protein S and C deficiency [4]. In a cross-sectional research conducted by Josephson et al., it was shown that 43 out of 95 children had antiphospholipid antibodies, and some of them had lower levels of protein S after being infected with VZV. The condition was referred to as varicella autoantibody syndrome [5].

CVST may manifest as a standalone condition characterized by increased pressure inside the skull, leading to symptoms such as headache, vomiting, and optic disc swelling (papilledema). Additionally, it might manifest as convulsions, changes in mental state, cranial nerve impairments, or localized neurological impairments [6]. The patient arrived at the emergency department with a headache, vomiting, fever, and a typical vesicular rash, all indicative of primary VZV infection.

Vasculopathies that occur after VZV infection are often identified by performing a cerebrospinal fluid polymerase chain reaction to detect anti-VZV immunoglobulin G antibodies and VZV deoxyribonucleic acid (DNA). Vasculopathy cannot be ruled out until both conditions are negative [1]. If there is suspicion of CVST, CT and MRI may be used to detect venous thrombosis or absence of blood flow in the cerebral veins, therefore confirming the diagnosis. Furthermore, it is necessary to assess protein S, protein C, and antithrombin III (AT-III) in order to exclude any previous hypercoagulable condition in patients with post-VZV CVST [7].

The diagnosis of post-primary VZV CVST was established based on clinical and radiological findings. During the examination in the emergency department, a CT scan detected a significant presence of blood clots in the dural sinuses, known as dural sinus venous thrombosis. Intravenous acyclovir is often used to treat virus-associated vascular problems [6]. In addition, the primary treatment for acute CVST often involves anticoagulant therapy with systemic heparin. Nevertheless, a small percentage (9%-13%) of patients using anticoagulant medication for CVST may have unfavorable results. This treatment may not effectively break the clot and, in rare cases, may even worsen the patient's clinical state.

The incidence of partial or full recanalization varied between 47% and 100% with anticoagulation alone. Other options for treating CVST include fibrinolytic medication, direct catheter thrombolysis, mechanical thrombolysis and thrombectomy, and surgical intervention. Anticoagulation treatment is often maintained after an acute CVST event [7]. After the diagnosis was confirmed, the patient received intravenous acyclovir and systemic heparin. Following the patient's clinical recovery, heparin was subsequently switched to warfarin for long-term treatment of venous thrombosis.

## Conclusions

It is important to be cautious and skeptical when encountering instances of post-varicella neurological symptoms since there exists a spectrum of neurological consequences associated with chicken pox and VZV vasculopathy. Swift and precise diagnosis may result in successful antiviral therapy of the VZV vasculopathy disease spectrum.
